# Impact of coprophagy prevention on the growth performance, serum biochemistry, and intestinal microbiome of rabbits

**DOI:** 10.1186/s12866-023-02869-y

**Published:** 2023-05-10

**Authors:** Zhitong Wang, Hui He, Mengjuan Chen, Mengke Ni, Dongdong Yuan, Hanfang Cai, Zhi Chen, Ming Li, Huifen Xu

**Affiliations:** 1 College of Animal Science and Technology, Henan Agricultural University, Zhengzhou, 450046 China; 2 College of Animal Science and Technology, Yangzhou University, Yangzhou, 225000 China

**Keywords:** Caecotrophy, Growth performance, Microbiome, SCFAs, Rabbits

## Abstract

**Background:**

Coprophagy plays a vital role in maintaining growth and development in many small herbivores. Here, we constructed a coprophagy model by dividing rabbits into three groups, namely, control group (CON), sham-coprophagy prevention group (SCP), and coprophagy prevention group (CP), to explore the effects of coprophagy prevention on growth performance and cecal microecology in rabbits.

**Results:**

Results showed that CP treatment decreased the feed utilization and growth performance of rabbits. Serum total cholesterol and total triglyceride in the CP group were remarkably lower than those in the other two groups. Furthermore, CP treatment destroyed cecum villi and reduced the content of short-chain fatty acids (SCFAs) in cecum contents. Gut microbiota profiling showed significant differences in the phylum and genus composition of cecal microorganisms among the three groups. At the genus level, the abundance of *Oscillospira* and *Ruminococcus* decreased significantly in the CP group. Enrichment analysis of metabolic pathways showed a significantly up-regulated differential metabolic pathway (*PWY-7315*, dTDP-N-acetylthomosamine biosynthesis) in the CP group compared with that in the CON group. Correlation analysis showed that the serum biochemical parameters were positively correlated with the abundance of *Oscillospira*, *Sutterella*, and *Butyricimonas* but negatively correlated with the abundance of *Oxalobacte* and *Desulfovibrio*. Meanwhile, the abundance of *Butyricimonas* and *Parabacteroidesde* was positively correlated with the concentration of butyric acid in the cecum.

**Conclusions:**

In summary, coprophagy prevention had negative effects on serum biochemistry and gut microbiota, ultimately decreasing the growth performance of rabbits. The findings provide evidence for further revealing the biological significance of coprophagy in small herbivorous mammals.

**Supplementary Information:**

The online version contains supplementary material available at 10.1186/s12866-023-02869-y.

## Introduction

Rabbit is one of the small herbivore mammals, that have developed and maintained the behavior of caecotrophy to improve their digestion efficiency during the long-term evolution. Owing to the characteristics of their digestive system, caecotrophy is necessary for them to maintain their normal growth and reproduction performance under adverse living environments by supplying the essential nutrients [1]. The cecum tissues of rabbits are strikingly complex and highly developed, rabbits expand the size of their cecum to further access the complex carbohydrates of plants [2]. In addition, the cecum is also an important fermentation organ of rabbits where indigestible lignin and cellulose can be decomposed through microbial fermentation [3, 4]. It has the highest abundance of microorganisms compared with other intestinal sites in rabbits [5]. These microbial communities have profound effects on the nutrition, physiology, and even behavior of rabbits [6, 7]. Recent studies pointed out that gut microbiota was implicated in the regulation of many physiological processes including digestion, neuroendocrine, and immune response [8–10]. However, the main function of gut microbiota is to ferment dietary fiber into short-chain fatty acids (SCFAs) [11], which plays an important role in chemotaxis and phagocytosis, cell proliferation, anti-inflammatory, and anti-tumorigenic [12]. SCFAs are also important nutrients for intestinal epithelial cells [13, 14]. Therefore, the diversity and homeostasis of intestinal flora is important in maintaining the health and reproduction performance of animals.

Coprophagy is common in many animals, including rats, termites, and rabbits [15–17]. Rabbits produce two types of feces: hard feces and soft feces [18, 19]. “Colonic separation mechanism” is important for the formation and excretion of both types of feces [20]. Compared with hard feces, soft feces contained more water, crude protein, total amino acids, essential amino acids, minerals (e.g., Na, Cl, K), and other nutrients [21]. Rats can fine tune their fecal intake according to their needs. One study demonstrated that rats deficient in thiamine and pantothenic acid increase their fecal intake [22]. The nutrient composition in soft feces and cecal contents is similar [23, 24]. Caecotrophy contributes to maintain energy balance and cognitive performance by stabilizing the gut microbiota and promoting microbial metabolism in Brandt’s vole [25]. And caecotrophy is involved in regulating the nutritional value of rabbit meat [26]. Therefore, coprophagy is of great biological importance in small herbivores.

Although caecotrophy behavior has received increasing attention, its effects on the growth performance, metabolism, intestinal morphology, and microecology of rabbits have not been fully investigated. Here, our hypothesis is that coprophagy prevention decreases the growth performance of rabbits by altering their metabolism and cecal microbiome. To verify this hypothesis, we constructed a coprophagy prevention model to explore the effects of coprophagy prevention on the growth performance, gut morphology, gut microbiota, and blood biochemical indexes of rabbits. The results provide evidence to further clarify the biological significance of coprophagy and contribute to the production of healthy and high-quality rabbit meat.

## Results

### Coprophagy prevention decreased the growth performance of rabbits

**Fig. 1:**
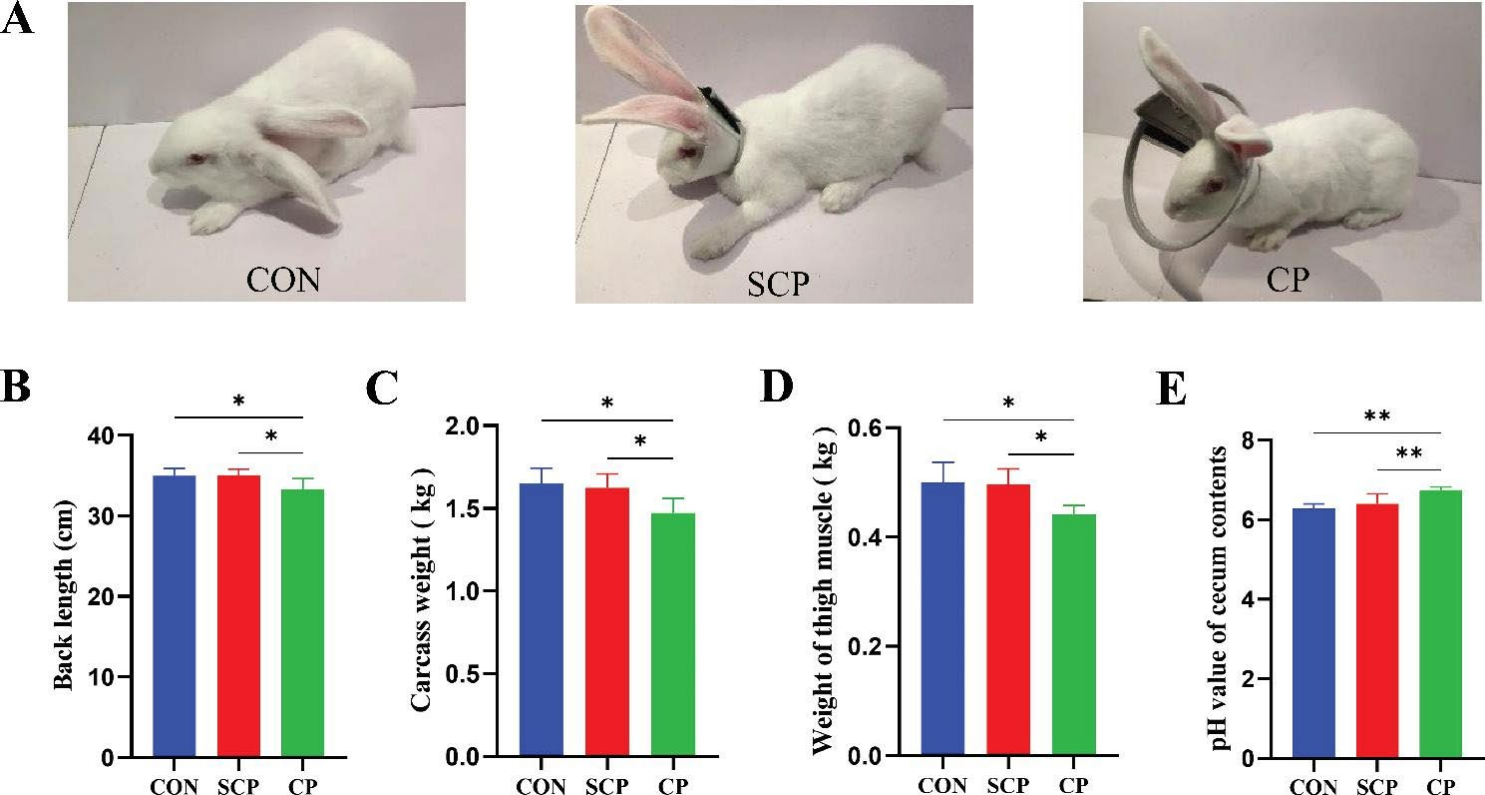
Effects of coprophagy prevention on growth phenotype of rabbits. Rabbits in CON, SCP and CP group **(A)**; back length (BL) **(B)**, carcass weight **(C)**, weight of thigh muscle **(D)** and pH value of cecum contents **(E)** of CON, SCP and CP groups. (CON = control, SCP = sham-coprophagy prevention, CP = coprophagy prevention; * *P* < 0.05; ** *P* < 0.01 )

As shown in Table 1 and Supplementary Fig. S1, no significant difference in initial body weight and daily feed intake was observed among the three groups (*P* > 0.05). The slaughter weight and ADWG of the CP group’s rabbits were significantly lower than those of the CON and SCP groups (Table 1, *P* < 0.05), resulting in a significant increase in the FCR of the CP group relative to that of the other two groups (Table 1, *P* < 0.05). At the end of the experiment, the carcass traits of rabbits were measured after slaughter. Our results showed that the back length, carcass weight and thigh muscle weight in the CP group were significantly lower than those in the other two groups (Fig. 1B C, and 1D, P < 0.05). The pH of the cecum content of the CP group was significantly higher than that of the other two groups (Fig. 1E, P < 0.01).

**Table 1:** Effects of coprophagy prevention on growth phenotype of rabbits

Parameter	Group		
	CON	SCP	CP
Initial body weight (kg)	1.80 ± 0.18	1.83 ± 0.11	1.82 ± 0.19
Slaughter weight (kg)	3.18 ± 0.18^a^	3.19 ± 0.15^a^	2.94 ± 0.17^b^
Average daily weight gain (g/d)	33.56 ± 3.35^a^	32.51 ± 4.11^a^	24.69 ± 4.25^b^
Feed conversion rate	6.84 ± 0.84^b^	6.89 ± 0.97^b^	9.34 ± 1.30^a^

^ab^ Means in the same row with different superscript letters differ significantly at 0.05

### Effects of coprophagy prevention on serum biochemistry, cecum structure and SCFAs in cecum contents

**Fig. 2:**
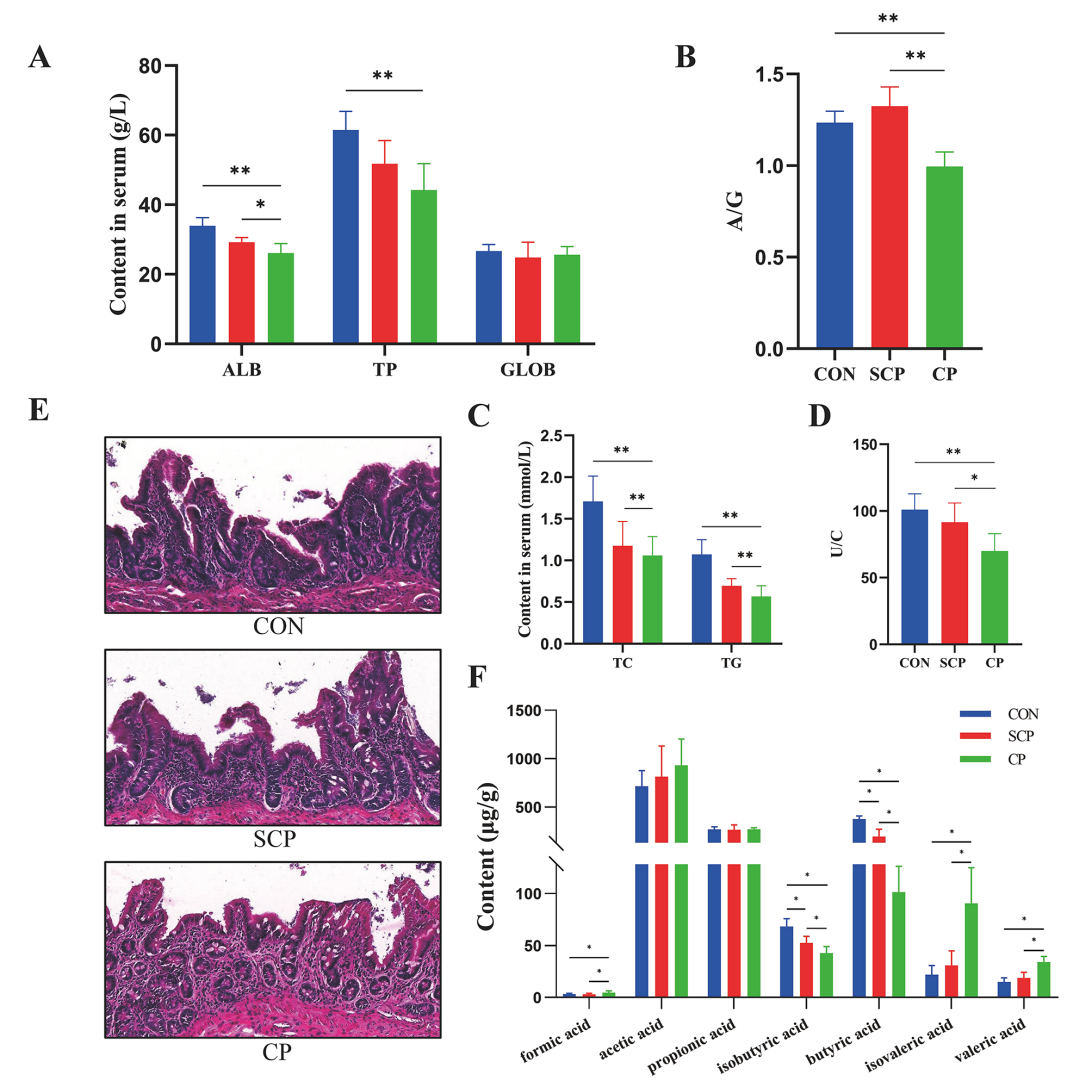
Effects of coprophagy prevention on serum biochemistry, cecum structure and SCFAs of cecum contents in rabbits. Albumin (ALB), total protein (TP), globulin (GLOB) **(A)**; albumin to globulin ratio (A/G) **(B);** total cholesterol (TC), triglycerides (TG) **(C);** and the ratio of urea nitrogen to creatinine (U/C) **(D);** hematoxylin-eosin staining of cecum sections **(E);** SCFAs in cecum contents **(F)**. (CON = control, SCP = sham-coprophagy prevention, CP = coprophagy prevention; * *P* < 0.05; ** *P* < 0.01)

Following coprophagy prevention, the ALB, TP and A/G ratio of the CP group were significantly lower than those of the CON group (Fig. 2A, B and P < 0.01). No significant difference in GLOB was fond among the three groups (Fig. 2A, P > 0.05). The TC and TG of the CP group were significantly lower than those of the CON group and SCP group (Fig. 2C, P < 0.01). In addition, the serum ratio of urea nitrogen to creatinine of the CP group was significantly lower than that of the CON group (Fig. 2D, P < 0.01) and SCP group (Fig. 2D, P < 0.05).

By measuring the microstructure of rabbits’ cecum, we found that coprophagy prevention caused severe damage to cecum villi relative to those of the CON group and SCP group (Fig. 2E). GC/MS was used to determine the content of cecum SCFAs to further explore the effect of coprophagy prevention on cecum microecology. The results suggested that the content of formic acid, isovaleric acid, and valeric acid in the cecum of the CP group were significantly higher than that in the other two groups (*P* < 0.05). However, CP treatment significantly decreased the content of isobutyric acid and butyric acid in cecum relative to that in the other two groups (Fig. 2F, P < 0.05).

### Alpha and beta diversity in the microbiota of CON, SCP, and CP groups

**Fig. 3:**
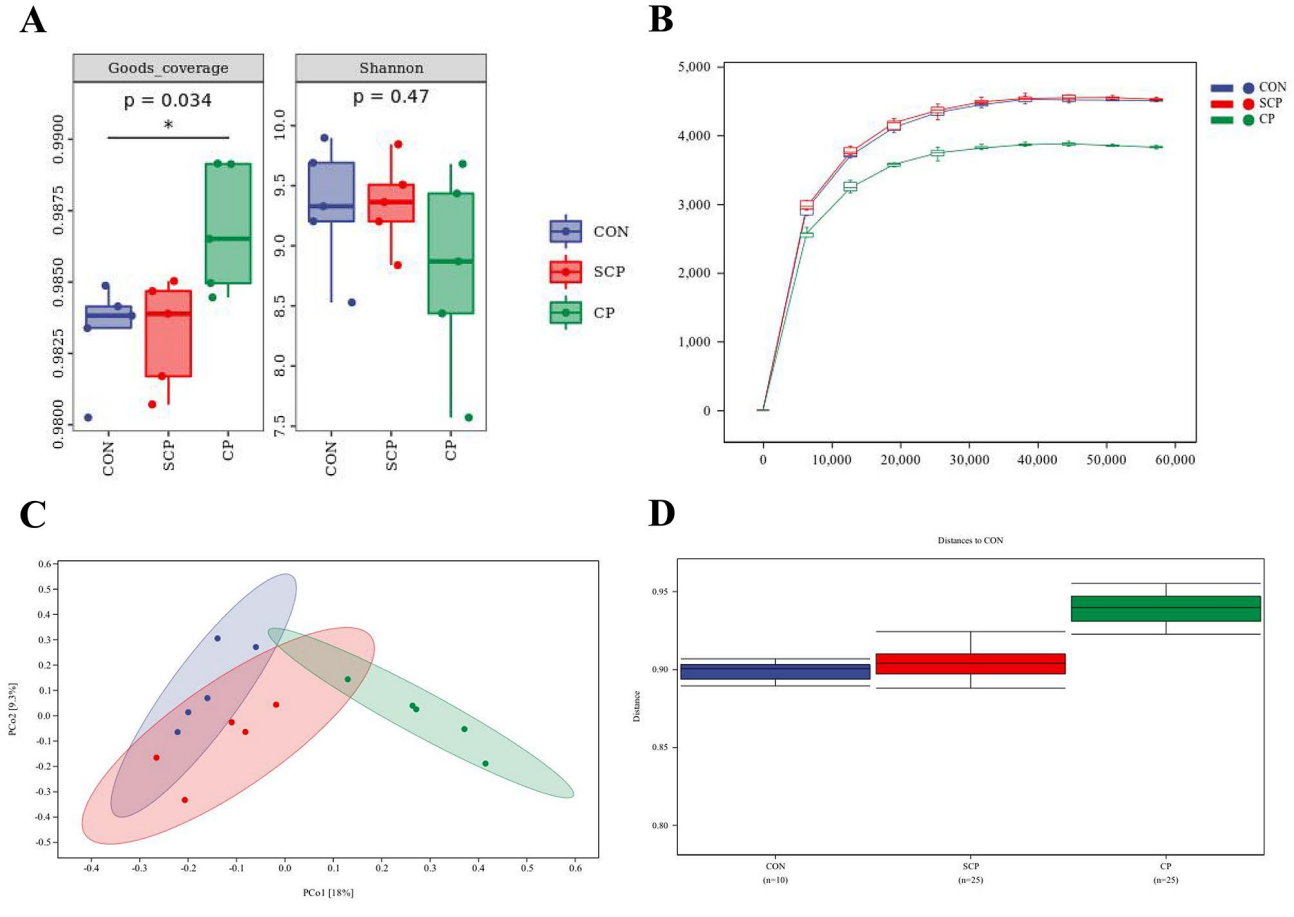
Effects of coprophagy prevention on the Alpha-diversity and Beta-diversity of cecum microorganisms in rabbits. Boxplots showed alpha-diversity based on goods’s coverage index and shannon index **(A);** Rarefaction curve based on chao1 of CON, SCP and CP groups **(B);** Principal coordinates analysis (PCoA) plot of beta-diversity based on bray’s curtis **(C);** analysis of similarities based on jaccard of CON, SCP and CP groups **(D)**. Each dot in the graph represents a sample, and dots of different colors indicate different groups. (CON = control, SCP = sham-coprophagy prevention, CP = coprophagy prevention; * *P* < 0.05; ** *P* < 0.01; different lowercase indicates significant differences in the same column *P* < 0.05)

The Good’s coverage of the three groups were higher than 98% (Fig. 3A). Shannon index showed that the bacterial community diversity in the CON and SCP groups was higher than that in the CP group (Fig. 3A). Sparse curve results for Chao1 index are shown in Fig. 3B. PCoA analysis showed that the microbial composition of the CP group was significantly different from that of the other two groups based on Bray’s Curtis (Fig. 3C). Permutational multivariate analysis of variance showed that the microbiota was significantly different between the CP and CON groups (*P* < 0.05) and between the CP and SCP groups (*P* < 0.01) based on Jaccard (Fig. 3D).

### Effects of coprophagy prevention on the cecum microbiota of rabbits

**Fig. 4:**
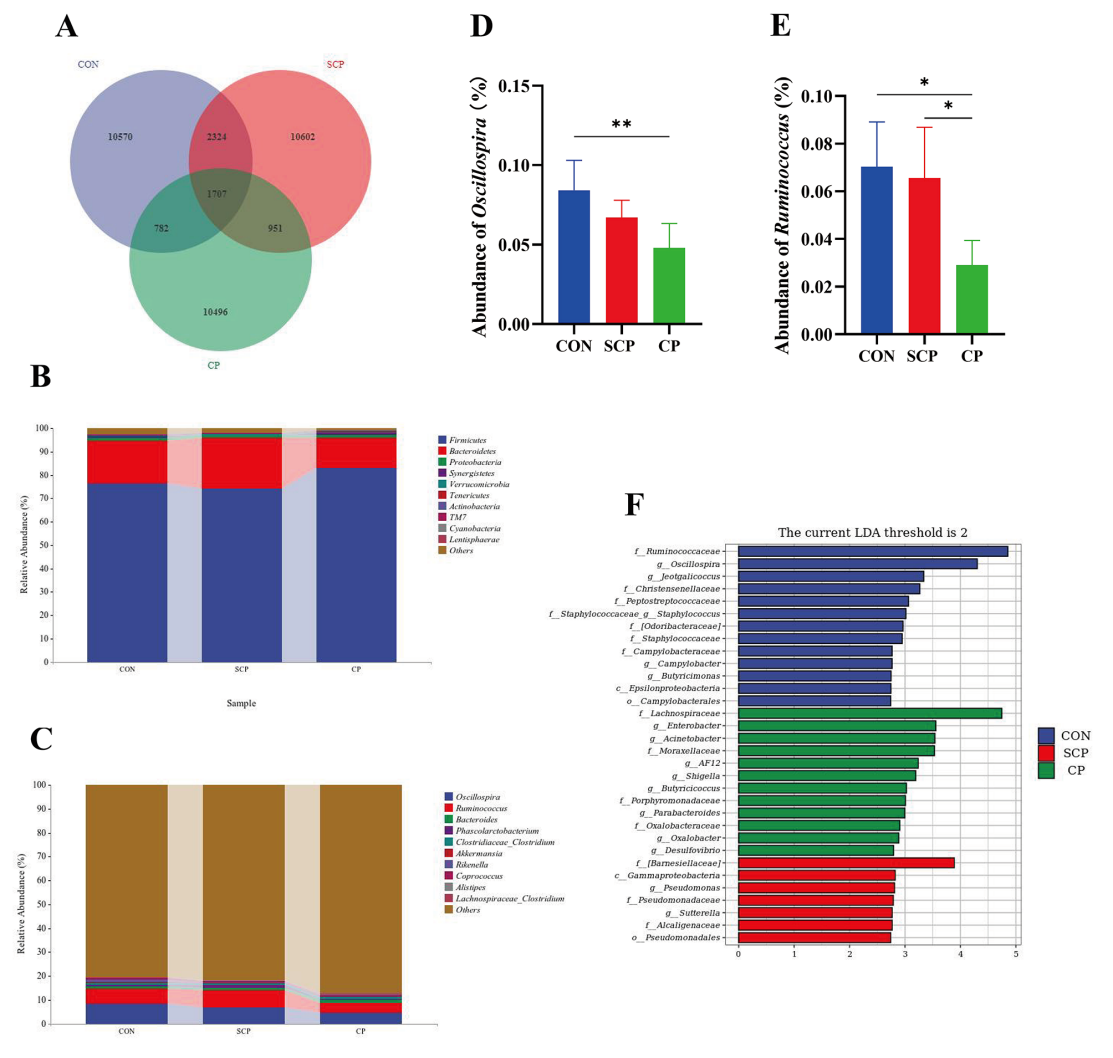
Effects of coprophagy prevention on cecum microbiota of rabbits. The Venn diagram summarizing the numbers of common and unique ASVs in the microflora community **(A);** distribution of phylum **(B)** and genus **(C)** levels for all sample microorganisms; the relative abundance of *Oscillospira* (D); the relative abundance of *Ruminococcus***(E);** LEfSe analysis based on Linear discriminant analysis **(F)**. (CON = control, SCP = sham-coprophagy prevention, CP = coprophagy prevention; * *P* < 0.05; ** *P* < 0.01 )

The Venn diagram showed 1,707 common ASVs in the cecum of the three groups of rabbits (Fig. 4A). A total of 10,570, 10,620, and 10,496 unique ASVs were identified in the CON, SCP, and CP groups, respectively. In addition, the top 10 differential flora of the rabbits’ cecum among the three groups were measured at the phylum level and the genus level. At the phylum level, the dominant phyla were Firmicutes, Bacteroidetes, Proteobacteria, Synergistetes, Verrucomicrobia, Tenericutes, Actinobacteria, TM7, Cyanobacteria and Lentisphaerae (Fig. 4B). At the genus level, the dominant microflora were *Oscillospira*, *Ruminococcus*, *Bacteroides*, *Phascolarctobacterium*, *Clostridiaceae_Clostridium*, *Akkermansia*, *Rikenella*, *Coprococcus*, *Alistipes* and *Lachnospiraceae_Clostridium* (Fig. 4C). The relative abundance of *Oscillospira* in the CP group was significantly lower than the CON group (Fig. 4D, P < 0.01), and the relative abundance of *Ruminococcus* in the CP group was significantly lower than that in the other two groups (Fig. 4E, P < 0.05). The results of LEfSe analysis (LDA > 2, *P* < 0.05) are shown in Fig. 4F. At the genus level, *Oscillospira*, *Jeotgalicoccus*, *Staphylococcus*, *Campylobacter* and *Butyricimonas* were abundant in the CON group at the genus level; while *Enterobacter*, *Acinetobacter*, *AF12*, *Shigella*, *Butyricicoccus*, *Parabacteroides*, *Oxalobacter* and *Desulfovibrio* were enriched in the CP group; *Pseudomonas* and *Sutterella* were enriched in the SCP group.

### Functional prediction of microbiota

**Fig. 5:**
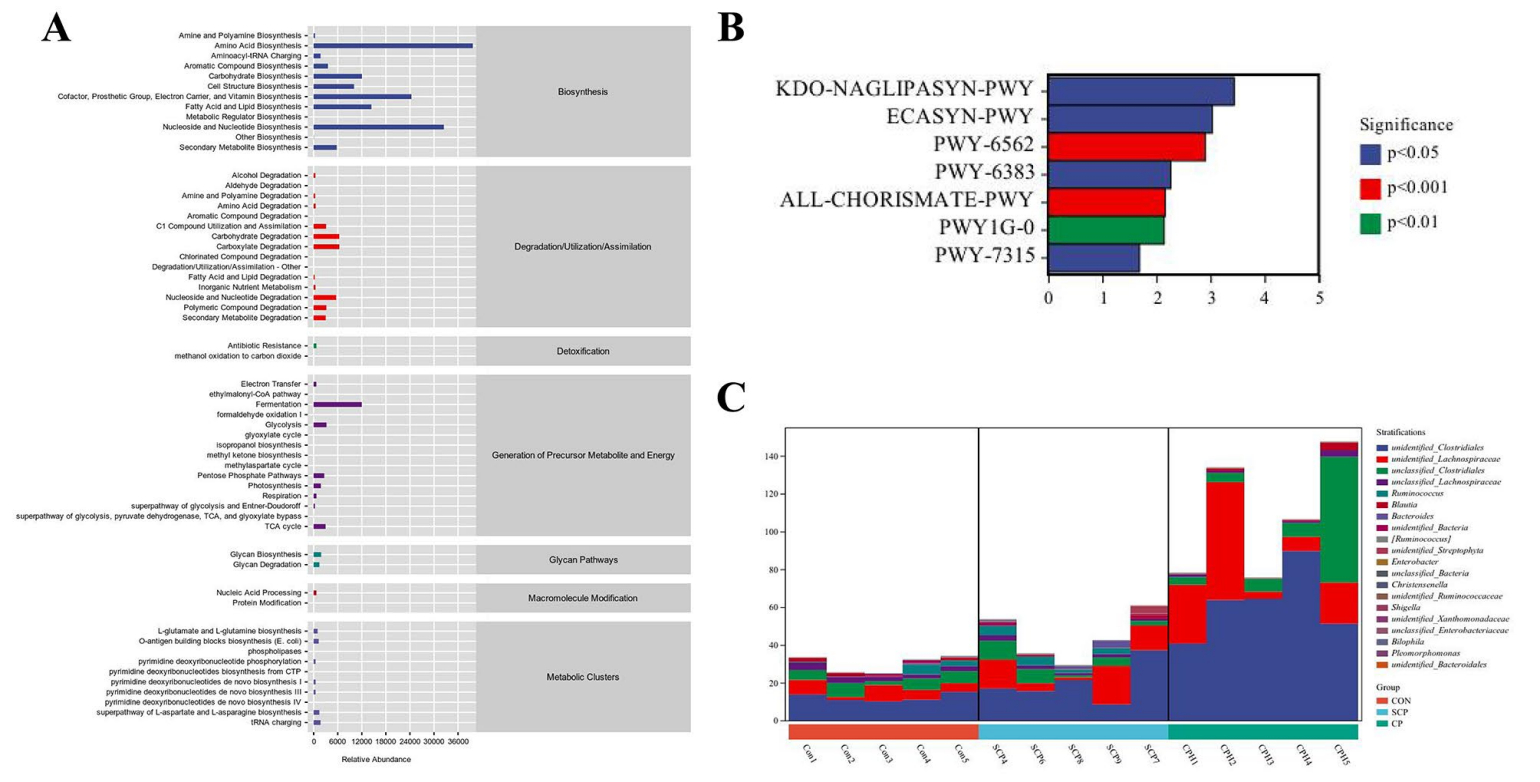
Functional potential prediction of microbiota. The analysis results of metacyc metabolic pathway including various pathways involved in primary and secondary metabolism **(A)**, differential metabolic pathways **(B)**, microbial composition of differential metabolic pathways **(C)** of CON, SCP and CP groups. (CON = control, SCP = sham-coprophagy prevention, CP = coprophagy prevention; * *P* < 0.05; ** *P* < 0.01)

The relative abundance results of primary and secondary metabolic pathways in Metacyc database are shown in Fig. 5A. Our findings showed the relatively high abundance of pathways related to biosynthesis, these pathways included amino acid biosynthesis, nucleoside and nucleotide biosynthesis, fatty acid, and lipid biosynthesis, etc. We further identified the metabolic pathways with significant differences among the groups based on metagenome-Seq method. Compared with the CON group, the up-regulated metabolic pathway in the CP group included *KDO-NAGLIPASYN-PWY*: superpathway of (Kdo) 2-lipid A biosynthesis (*P* < 0.05), *ECASYN-PWY*: enterobacterial common antigen biosynthesis (*P* < 0.05), *PWY-6383*: mono-trans, poly-cis decaprenyl phosphate biosynthesis (*P* < 0.05), *PWY-7315*: dTDP-N-acetylthomosamine biosynthesis (*P* < 0.05), *PWY-6562*: norspermidine biosynthesis (*P* < 0.001), *ALL-CHORISMATE-PWY*: superpathway of chorismate metabolism (*P* < 0.001), *PWY1G-0*: mycothiol biosynthesis (*P* < 0.01) (Fig. 5B). By associating the pathways with species and analyzing species composition enriched in the *PWY-7315* pathway, we found that the relative abundance of *unidentified _ Clostridiales* and *unidentified _ Lachnospiraceae* in the CP group was higher than that in the CON group (Fig. 5C).

### Correlation analysis

**Fig. 6:**
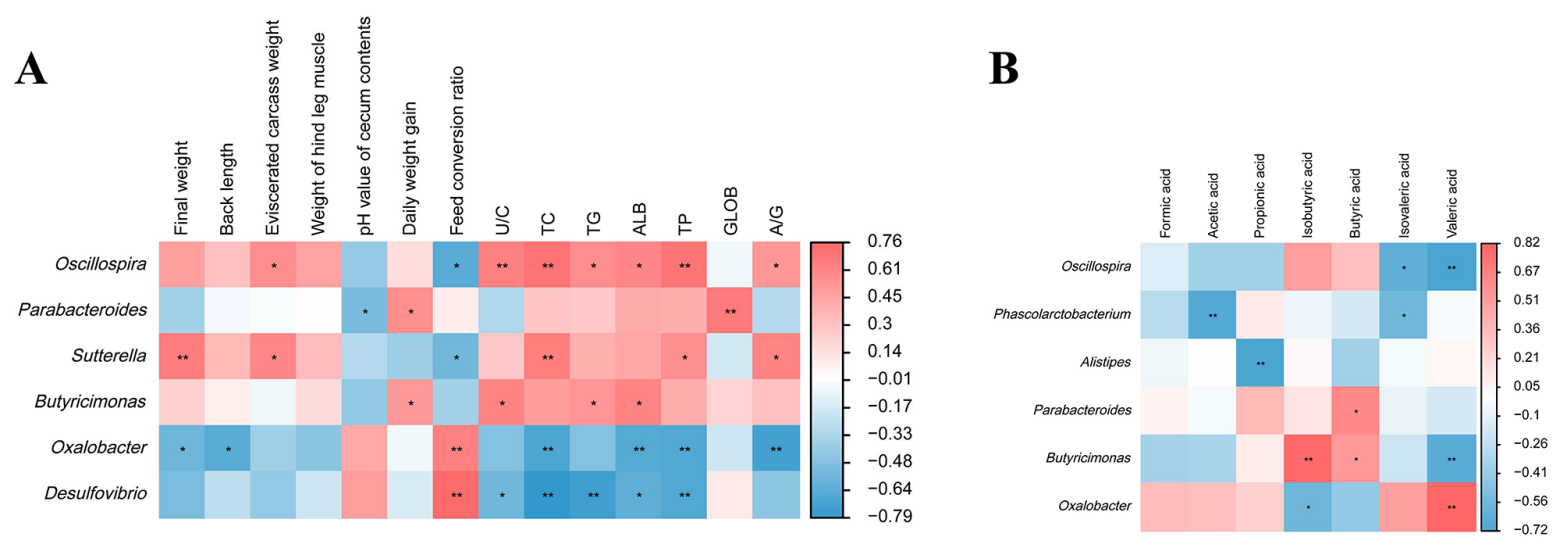
Correlation analysis of growth phenotype, serum biochemistry, SCFAs and microbiota. Correlation analysis of relative abundance of top 20 genera with growth phenotype and serum biochemistry in CON, SCP and CP groups **(A)**; correlation analysis of relative abundance of top 20 genera with SCFAs in CON, SCP and CP groups **(B)**. (CON = control, SCP = sham-coprophagy prevention, CP = coprophagy prevention; * *P* < 0.05; ** *P* < 0.01)

The results of the correlation analysis of growth phenotype, serum biochemistry, and microbiota are shown in Fig. 6A. The relative abundance of *Oscillospira* and *Sutterella* was positively related with ECW, U/C, TC, TG, ALB, TP and A/G, while negatively correlated with FCR. The relative abundance of *Butyricimonas* was positively correlated with DWG, UC, TG and ALB. The relative abundance of *Oxalobacte* was negatively correlated with FW, TC, ALB, TP and A/G but positively correlated with FCR. The relative abundance of *Desulfovibrio* was significantly negatively correlated with U/C, TC, TG, ALB and TP but significantly positively correlated with FCR.

The correlation analysis results of SCFAs and microbiota are shown in Fig. 6B. The relative abundance of *Oscillospira* was negatively correlated with isovaleric acid and valeric acid, that of *Alistipes* was negatively correlated with proplonic acid, and that of *Butyricimonas* was negatively correlated with valeric acid. In addition, the relative abundance of *Parabacteroides* and *Butyricimonas* was positively correlated with butyric acid, and that of *Oxalobacte* was positively correlated with valeric acid.

## Discussion

Caecotrophy is a common behavior in rabbits and many other small herbivores. The way of rabbit eating feces is similar to that of voles, they extend their mouth to the anus by turning back, and then directly swallow the soft feces [27, 28]. In these small herbivores, this behavior is of great biological importance by improving their digestive function, because many nutrients excreted by them are not fully digested and can be absorbed through eating feces [1, 29]. For example, rabbits and many herbivores can obtain essential amino acids and vitamins through caecotrophy [20, 30]. S Combes found that caecotrophy is important for rabbits to maintain intestinal homeostasis by replenishing the intestinal flora in soft feces [31]. In addition, this behavior also plays an important role in maintenance the gut microbiota in rats [20]. Therefore, caecotrophy behavior is of great biological importance for these small herbivores including rabbits. In the present study, New Zealand white rabbits were used to investigate the effects of coprophagy on growth performance and cecum microecology. Our previous studies reported that coprophagy prevention reduces the growth and reproduction performance of rabbits [32, 33]. The present study further demonstrated that CP treatment could reduce rabbits’ TC, TG, and other serum biochemical indicators; caused damage to the cecum villi; and reduced isobutyric and butyric acid levels in the cecum contents. Given the above results, 16S rRNA microbiome was performed to study the differences in cecum microbial communities under CP. We found that CP can decrease the relative abundance of *Oscillospira* and *Ruminococcus*.

The production efficiency of rabbits is high for their short growth and reproduction cycle [34]. Rabbit meat is healthy for its special nutritional properties, such as high content of polyunsaturated fatty acids, proteins, and essential amino acids [35]. These characteristics enable rabbit meat to satisfy the desire of modern consumers for a healthy lifestyle [36]. Therefore, research focused on the improving the growth and reproductive performance of rabbits is of great economic importance. Our results showed that CP increased FCR and reduced slaughter weight, which are consistent with previous studies in rats [37]. The soft feces excreted by rodents are rich in crude protein, total amino acids, essential amino acids, minerals and other nutritional elements [18, 33]. CP treatment decreases the total nutrient intake of rodent feces [38]. Therefore, we speculated that the decrease of growth performance in CP group’s rabbits is caused mainly by the fact that CP treatment reduces the reabsorption of nutrients from soft feces in rabbits.

Serum biochemistry and cecal histomorphology further confirmed this speculation. Serum biochemical indexes can be used to evaluate the health status of livestock. Although this study showed that serum TC and TG were reduced by CP treatment, previous reports indicated that high-fat feeding can increase the content of serum TC and TG [39, 40]. This difference may be due to the weakened nutrient absorption of the CP group, which is consistent with our growth performance results. Our finding showed that CP destroyed the mucosal layer of cecum and reduced the villus length of cecum. The villus length of cecum is positively correlated with nutrient absorption efficiency in rabbits [41, 42]. Damage to cecal mucosa is also associated with the production of SCFAs, especially butyrate [43]. SCFAs exert multiple beneficial effects on various aspects of mammalian energy metabolism [11]. Previous studies have shown that butyric acid regulated intestinal inflammation and affected the intestinal mucosa [44]. Intestinal inflammation is closely related to the immune status of the body. It has been demonstrated that reduced levels of serum TP and ALB indicate an immune imbalance in the body [45]. In our study, serum TP and ALB were significantly lower in the CP group than in the other two groups, which may be related to intestinal damage as well as immune imbalance in rabbits. By using GC-MS analysis, we found that CP treatment reduced the content of butyric acid in cecum. Reduction of butyric acid may cause inflammation and destruction of the cecum mucosa.

The rapid development of high-throughput sequencing technology helps researchers to further understand the composition of host gut microbiota [46]. Researchers can identify key regulatory metabolic pathways and determine key microbiota through combined microbiome and metabolome analysis [47]. Given that the cecum is the main site of crude fiber digestion in rabbits and contains a rich diversity of bacteria [19], the contents of the cecum were collected for 16S rRNA gene sequencing. Our results showed that CP treatment reduced the relative abundance of *Oscillospira* and *Ruminococcus*. Previous studies have shown that *Ruminococcus* is important in regulating the production of intestinal mucus [48]. Intestinal mucus is mainly produced by goblet cells and is found in the intestinal villi. Intestinal mucus not only protects intestinal health but also contains many immunomodulatory molecules [49, 50]. Furthermore, studies have shown that intestinal mucus plays an important role in regulating the transportation of nutrients [51]. Thus, a reduction in the relative abundance of *Ruminococcus* will affect gut morphology, immunity, and nutrient absorption. *Oscillospira* may produce butyrate [52], which is in agreement with the result of SCFAs in this study. All these findings implied that CP treatment decreased the growth and development of rabbits by regulating the intestinal microflora and intestinal SCFAs content. However, this study only evaluated the growth performance and serum biochemistry, intestinal microbiome changes caused by CP in rabbits, the underlying mechanism need to be explored via molecular approaches. In addition to its nutritional value, whether caecotrophy has other biological implications remains to be further studied. Findings in this study will provide reference and insight for further studying the biological importance of caecotrophy in small herbivores.

## Materials and methods

### Animal ethics

This study was designed and carried out in accordance with the guidelines of the Institutional Animal Care and Use Committee (No. 11–0085) of the College of Animal Science and Technology, Henan Agricultural University, China.

### Animal feeding and sample collection

A total of 15 healthy New Zealand white Rabbits (male, 46-day-old) with the similar body weight (1.79 ± 0.21 kg) were provided by Mengfei Rabbit Breeding Co., Ltd (Zhengzhou, China). These rabbits were randomly divided into three groups (n = 5): control group (CON), sham-coprophagy prevention group (SCP) and coprophagy prevention group (CP). As shown in Fig. 1A, rabbits in the CON group were fed normally without collar, while rabbits in SCP group were fitted with a narrow collar (5.0 cm width) that did not prevent rabbits from coprophagy, and rabbits in the CP group were treated with a wide collar (8.5 cm width) that prevent rabbits from coprophagy. Each rabbit was reared in a separate cage. Feed intake was recorded daily and body weight was measured weekly for each rabbit. The amount of pellet feed added and remaining was recorded daily. The ingredient and nutrient composition of the granular diets used in this study were shown in Supplementary Table. S1. Rabbits were provided ad libitum access to water and granular diets. The pretrial period was 7 days and the experimental period was 42 days.

At the end of this experiment, rabbits were treated with food and water deprivation for 24 h. Then blood sample from ear veins was collected before slaughter, and serum was collected immediately by 10 min of centrifugation at 3 000 rpm at 4 °C. The obtained serum sample was stored at -80 °C for blood biochemical indexes analysis. Then rabbits were anesthetized with an overdose of isoflurane (Abbot, Chicago, IL, USA) and then slaughtered for sample collection. The cecum content of each rabbit was immediately collected and stored at -80 °C. The caecum tissue (3 cm length) used for HE staining was collected and immediately fixed in 4% paraformaldehyde, followed by fixation at 4 °C for 24 h.

### Growth performance measurement

The initial body weight of each rabbit was weighed at the beginning of the experiment. At the end of the experiment, the slaughter weight and back length of each rabbit was measured. The amount of feed added and the amount of residual feed in each rabbit cage was recorded daily. Total feed intake (TFI), average daily weight gain (ADWG) and feed conversion rate (FCR) were calculated. ADWG was the ratio of total weight gain (g) to total feeding days (d) of rabbits. FCR was the ratio of total feed intake (kg) to total weight gain (kg) of rabbits [53].

### Carcass traits

Slaughter and carcass dissection performance are evaluated according to the World Rabbit Science Association standards [54]. After slaughter, rabbits were first bled and skinned, followed by immediately removal of viscera, fat, head (head disconnected from the atlantoaxial region), forelimbs (disconnected along the wrist joint) and hindlimbs (disconnected along the tarsal joint). The stuff, remained on the carcass, which were blood stains, furs and others were removed by clean gauze. We then removed the hind legs from the carcass and dissected the thigh muscles. Finally, carcass weight and weight of thigh muscle were weighed.

### Determination of pH of cecum contents and preparation of cecum sections

The pH meter (Testo205, Testo, Germany) was inserted into the cecum content to determine the pH value. Each rabbit was measured three times and then averaged.

Haematoxylin and eosin (HE) staining of cecum was performed according to a previously reported method by Servicebio Co., Ltd. (Wuhan, China) [55]. The cecum tissues of rabbits were embedded in paraffin. Serial Sect. (5-µm thick) were dewaxing with xylene and hydrating in an ethanol series of descending concentrations, then subjected for Haematoxylin-Eosin (HE) staining. These slices were observed under the Nikon optical microscope (Nikon Eclipse E100, Tokyo, Japan). Visualization was performed under optical microscope (Eclipse Ci-L, Nikon, Japan).

### SCFAs analysis of cecum content

The content of SCFAs was analyzed using gas chromatography-mass spectrometry (GC-MS). The sample of cecum content was processed and analyzed according to a previously published method [56]. Briefly, 50 mg of cecum content was placed in a 2 mL EP tube, and extracted with 170 µL of ultra-pure water and 30 µL of disodium hydrogen phosphate (0.5 mol/L). Subsequently, 600 µL of 5% PFBBr solution was added to the extract and incubated for 40 min at 60 °C in a water bat. Then 200 µL of hexane was added for extraction, and the upper hexane phase was centrifuged at 6 000 rpm for 15 min, and the upper hexane phase was analyzed by GC-MS (7890B/5977A GC/MSD, Agilent, USA). Helium was used as the carrier gas. The initial temperature was maintained at 60 °C for 1 min; raised to 100 °C at a rate of 20 °C/min, then raised to 110 °C at a rate of 2 °C/min and kept for 4 min; raised to 160 °C at a rate of 15 °C/min, raised to 230 °C at a rate of 20 °C/min. Inlet temperature was 280 °C and transfer line temperature was 280 °C. Sample volume was 1 µL, front inlet septum purge flow was 3 mL/min. In the electronic shock mode, the energy was − 70 eV. After a solvent delay for 2 min, the MS data were obtained in full scan mode with 50 to 550 m/z range at a rate of 2 spectra per second.

### Serum biochemical assays

The serum biochemical indexes of rabbits were measured by using an animal biochemical analyzer (SMT-120VP, Seamaty, CHINA) and animal biochemical reagent plate (Seamaty, CHINA). The concentrations of albumin (ALB), total protein (TP), globulin (GLOB), ratio of albumin to globulin (A/G), the ratio of urea nitrogen to creatinine (U/C), total cholesterol (TC) and triglyceride (TG) in serum were measured in the present study.

### 16S rRNA gene amplicon sequencing

16S rRNA sequencing was performed by Personalbio (Shanghai, China). Fresh gut content collected from the cecum of rabbits were immediately frozen and stored at − 80 °C. Genomic DNA was extracted by using Genomic DNA Extraction Kit (Tian gen, Beijing, China) according to the instructions. PCR amplification of the bacterial 16S rRNA genes V3–V4 region was performed using the forward primer 338 F (5’-ACTCCTACGGGAGGCAGCA-3’) and the reverse primer 806R (5’-GGACTACHVGGGTWTCTAAT-3’). After purification, quantification, and equal mixing of PCR amplicons, the Illumina NovaSeq platform was used for sequencing.

The microbiome bioinformatics for this experiment were performed using QIIME2 2019.4 [57]. Briefly, raw sequence data was demultiplexed using the demux plugin and then primers cutting with cutadapt plugin. Quality filtering, denoising, merging, and chimera removal of sequences were performed using the DADA2 plugin [58]. The above sequences were grouped according to similarity to generate characteristic sequences ASVs, and the taxonomic information corresponding to each ASV was obtained by comparing the characteristic sequences of ASVs with the reference sequences in the database using the Greengenes database (Release 13.8, http://greengenes.secondgenome.com/). In this experiment, within-habitat diversity and between-habitat diversity was characterized using alpha diversity and beta diversity, respectively, in order to evaluate their overall diversity in an integrated manner. In this study, Good’s coverage index was used to represent coverage, and the Shannon index was used to represent diversity. The rarefaction curve reflected the impact of sequencing depth on the diversity of observed samples and the curves of samples tend to be gentle, indicating that the sequencing results were enough to reflect the diversity contained in the current samples. The PICRUSt2 (Phylogenetic Investigation of Communities by Reconstruction of Unobserved States) was used to predict the function of microorganisms upon the MetaCyc database (https://metacyc.org/) [59].

### Correlation analysis of growth phenotype, serum biochemistry, SCFAs and microbiota

The genescloud tools (https://www.genescloud.cn) were used to analyze the correlation of growth phenotype (slaughter weight, ADWG, FCR, BL, carcass weight, weight of thigh muscle, and pH value of cecum contents), serum biochemistry (U/C, TC, TG, ALB, TP, GLOB, and A/G), SCFAs and microbiota (the top 20 genera) among the three groups. Import data into the cloud platform for Spearman correlation analysis. The correlation analysis modeling was performed according to a previous study [60].

### Statistical analysis

A completely randomized trial design was used in this study. All treatments were performed in biological quintuplicate and data are presented as means ± SE. Statistical analysis was performed with independent sample t-test to compare the effects of coprophagy prevention by SPSS 24.0. * indicates significant difference, *P* < 0.05; ** indicates a very significant difference, *P* < 0.01; NS indicates that there is no significant difference between the data, i.e. *P* > 0.05.

## Conclusions

Our results enhanced the important role of coprophagy in maintaining the growth performance and intestinal healthy in rabbits. The dramatic changes of microbiota profile and SCFAs content caused by coprophagy prevention in cecum is tightly correlated with the growth performance and metabolic indicators of rabbits. Our study is helpful for further understanding the biological significance and regulatory mechanism of fecal eating behavior in rabbits and other small herbivores.

## Electronic supplementary material

Below is the link to the electronic supplementary material.

Supplementary Material 1

## Data Availability

The datasets generated and analyzed during the current study are available in the Sequence Read Archive (SRA) under the BioProject: PRJNA910808.
