# Supplementary material for: Impact of coprophagy prevention on the growth performance, serum biochemistry, and intestinal microbiome of rabbits
Source: BMC Microbiol. 2023 May 10;23:125. doi: 10.1186/s12866-023-02869-y (PMC10170819; doi:10.1186/s12866-023-02869-y)
Supplement: Supplementary file 1 — Supplementary Material 1 [file 12866_2023_2869_MOESM1_ESM.docx]

**Supplementary Table. S1 The composition and nutritional composition of diets (dry basis)**

| **Feeds** | **Contents (%)** | **Nutrient level** | **Contents (%)** |
| --- | --- | --- | --- |
| Soybean meal | 20.0 | DE (MJ/kg)^2)^ | 12.20 |
| Corn | 16.0 | Crude Protein | 14.88 |
| Wheat bran | 20.0 | Crude Fiber | 16.53 |
| Alfalfa meal | 15.0 | Ether extract | 2.64 |
| Peanut | 24.5 | Met | 0.65 |
| Soybean oil | 0.5 | Lys | 0.98 |
| Premix ^1)^ | 4.0 | Ca | 1.05 |
| Total | 100.0 | P | 0.35 |

1) Premix is VA 8000 IU, VD3 900 IU, VE 100 mg, VK3 2 mg, VB1 1 mg, VB2 3 mg, VB6 1 mg, VB12 0.01 mg, niacin 50 mg, pantothenic acid 8.0 mg, folic acid 0.5 mg, zinc 50mg, iron 50 mg, manganese 30 mg, magnesium 150 mg, iodine 0.5 mg, selenium 0.1 mg, salt 5 g, choline 1.5 g, methionine 3.0 g, lysine 2.9 g per kg of diet.

2) The digestible energy is the calculated value, and the rest is the measured value.

**
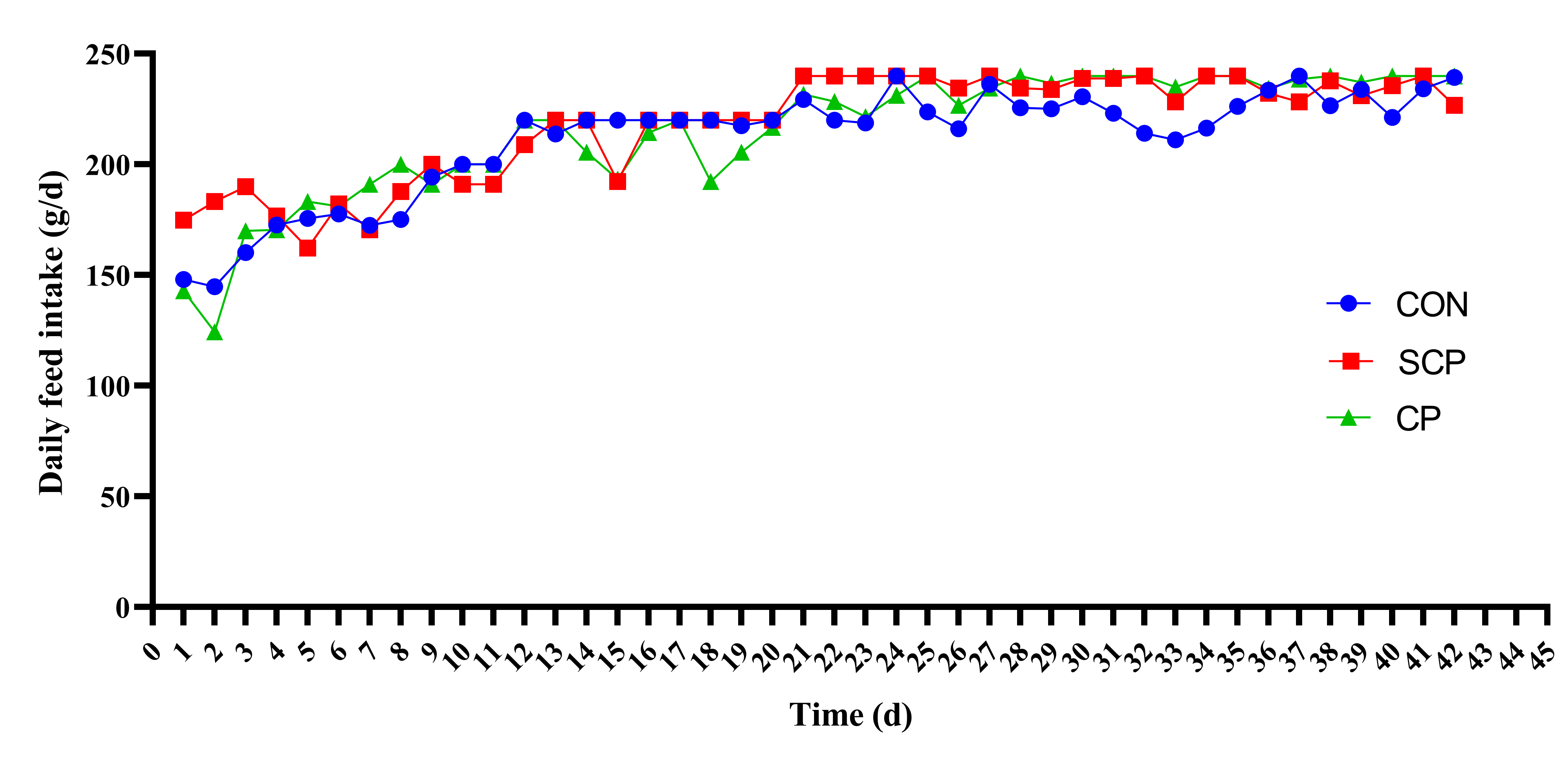
**

**Supplementary Fig. S1** Daily feed intake of rabbits in three groups.
